# A nonlinear associations of metabolic score for insulin resistance index with incident diabetes: A retrospective Chinese cohort study

**DOI:** 10.3389/fcdhc.2022.1101276

**Published:** 2023-01-12

**Authors:** Zhuangsen Chen, Caiyan Huang, Zhongyu Zhou, Yanrong Zhang, Mingyan Xu, Yingying Tang, Lei Fan, Kun Feng

**Affiliations:** ^1^ Department of Endocrinology, Pingshan District People’s Hospital of Shenzhen, Shenzhen, China; ^2^ Department of Endocrinology, Pingshan General Hospital of Southern Medical University, Shenzhen, China; ^3^ Department of Endocrinology, Heilongjiang Provincial Hospital, Harbin, China

**Keywords:** metabolic score for insulin resistance, insulin resistance, incident diabetes, relationship, nonlinear

## Abstract

**Background:**

The Metabolic score of insulin resistance (METS-IR) has recently been accepted as a reliable alternative to insulin resistance (IR), which was demonstrated to be consistent with the hyperinsulinemic-euglycemic clamp. Few pieces of research have focused on the relationship between METS-IR and diabetes in Chinese. The purpose of this research was to explore the effect of METS-IR on new-onset diabetes in a large multicenter Chinese study.

**Methods:**

At the baseline of this retrospective longitudinal research, 116855 participators were included in the Chinese cohort study administered from 2010 to 2016. The subjects were stratified by quartiles of METS-IR. To assess the effect of METS-IR on incident diabetes, the Cox regression model was constructed in this study. Stratification analysis and interaction tests were applied to detect the potential effect of METS-IR and incident diabetes among multiple subgroups. To verify whether there was a dose-response relationship between METS-IR and diabetes, a smooth curve fitting was performed. In addition, to further determine the performance of METS -IR in predicting incident diabetes, the receiver operating characteristic curve (ROC) was conducted.

**Results:**

The average age of the research participators was 44.08 ± 12.93 years, and 62868 (53.8%) were men. METS-IR were significant relationship with new-onset diabetes after adjusting for possible variables (Hazard ratio [HR]: 1.077; 95% confidence interval [CI]: 1.073-1.082, *P* < 0.0001), the onset risk for diabetes in Quartile 4 group was 6.261-fold higher than those in Quartile 1 group. Moreover, stratified analyses and interaction tests showed that interaction was detected in the subgroup of age, body mass index, systolic blood pressure, diastolic blood pressure, and fasting plasma glucose, there was no significant interaction between males and females. Furthermore, a dose-response correlation was detected between METS-IR and incident diabetes, the nonlinear relationship was revealed and the inflection point of METS-IR was calculated to be 44.43. When METS-IR≥44.43, compared with METS-IR < 44.43, the trend was gradually saturated, with log-likelihood ratio test *P* < 0.001. Additionally, the area under receiver operating characteristic of the METS-IR in predicting incident diabetes was 0.729, 0.718, and 0.720 at 3, 4, and 5 years, respectively.

**Conclusions:**

METS-IR was correlated with incident diabetes significantly, and showed a nonlinear relationship. This study also found that METS-IR had good discrimination of diabetes.

## Background

Diabetes mellitus (DM) is a chronic epidemic on an unprecedented scale, which is spiraling out of control (1). The International Diabetes Federation (IDF) reported that more than one in 10 adults now have diabetes all over the world. It is forecasted that 537 million people were suffered from DM in 2021. The Western Pacific region accounts for more than a third (38%) of the total number of diabetes, with China accounting for a quarter of the total (2, 3). In recent years, the Chinese Diabetes Society (CDS) has been paying attention to the progression of diabetes in China. The prevalence of diabetes in China is still on the rise, reaching 11.2% in 2017 (4, 5). It is well known that if diabetes is not well managed and treated, this disease will cause damage to multiple organs of the body and leads to complications, such as cardiovascular disease, kidney damage, eye disease, and so on. The direct and indirect costs of diabetes to health services increase continuedly (3, 6). It is a huge challenge to predict future diabetes incidence.

Insulin resistance (IR) is connected with the development and progression of diabetes significantly (7–9). It reduces insulin efficiency in insulin-responsive tissues (muscle, fat, and liver), which conversely causes a decompensated increase in insulin and hyperinsulinemia, and then leads to chronic metabolic disorders (hyperglycemia, hypertension, hyperlipidemia, etc.) and inflammation (10–14). Therefore, it is requisite to detect IR in the early stages of diabetes, and early prevention in non-diabetes patients with metabolic risk is beneficial to reduce the socioeconomic burden of diabetes and other metabolic diseases (15–18).

Accurate assessment of insulin resistance is usually performed with the Euglycaemic-hyperinsulinaemic clamp (EHC) and Homeostatic model assessment for insulin resistance (HOMA-IR). EHC is still the gold standard method of assessing IR. However, it is mostly utilized in research due to the characteristics of being time-consuming, invasive, and high cost, which inhibit its promotion (19–21). Meanwhile, the practical applicability of HOMA-IR is also limited by invasiveness, complexity, and impracticality, especially in resource-poor areas (21, 22). To evaluate IR in large epidemiological studies, several simple formulas have been developed based on readily available and inexpensive biochemical indicators. For example, triglyceride to high-density lipoprotein cholesterol ratio (TG/HDL-C), triglyceride glucose index (TγG index), and triglyceride glucose-body mass index(TγG-BMI) were widely employed as a reliable surrogate marker to estimate IR in clinical practice (23–28). These non-insulin-based indexes offer a simpler and cost-effective option for the identification of IR.

Recently, the Metabolic score for insulin resistance (METS-IR), a newly-developed non-insulin-based metabolic score, consists of fasting plasma glucose (FPG), triglycerides (TG), high-density lipoprotein cholesterol (HDL-C), and body mass index (BMI). It has been demonstrated to be highly consistent with EHC in assessing IR (29).

The relationship between METS-IR and the prevalence of diabetes, however, has received very little investigation, which needs to be further investigated, especially in large epidemiological studies. This research was designed to explore the effect of METS-IR on the incidence of diabetes among a large-scale of Chinese adults.

## Methods

### Cohort population and study design

Data for this research were collected from a multicenter health check-up program, the Rich Healthcare Group, which was published by Chen et al. at www.Datadryad.org (30). Data on this website is free and public, the providers give all copyright and ownership rights, and there is no interest involved. The detailed research design has been described in previous studies (30). Participants in this cohort underwent two or more follow-up visits between 2010 and 2016 in 11 major Chinese cities, and they were at least 20 years old. At each follow-up visit to the health check center, participants completed a detailed questionnaire and had blood samples collected. According to the report of Chen et al., 211,833 subjects (54.8% males and 45.2% females) were involved in this study (30). Data with missing values (such as weight, height, gender, and FPG) and with an extreme value of BMI (<15 kg/m2 or >55 kg/m2) were excluded at baseline. In addition, participants with a follow-up interval of fewer than 2 years, those who had been diagnosed with diabetes at baseline, and those who could not determine their diabetes status during follow-up were excluded. There was no need to apply for ethical approval due to the secondary data analysis nature of this study. Previous research received approval from the Rich Healthcare Group review board, and this retrospective study conformed with the Helsinki Declaration.

The dataset contains participants’ medical records, which includes demographic and blood biochemical variables: age, gender, height, weight, systolic and diastolic blood pressure (SBP and DBP), history of smoking, history of drinking, family history of the disease, FPG, TG, total cholesterol (TC), low-density lipoprotein cholesterol (LDL-C), HDL-C. BMI (kg/m^2^) is expressed as a weight (kg) divided by height squared (m^2^). Fasting venous blood samples were measured on an autoanalyzer, and participants need to be fasting for at least 10 hours. The dependent variable of the research was new-onset diabetes, defined as FPG≥7.00 mmol/L and/or self-reported diabetes during follow-up.

To further research, missing values and outliers of TG and HDL-C were excluded(n=94,978), and the total number of participants was 116,855 participants (62,868 males and 53,987 females) in the end. The arithmetic Formula of METS-IR was Ln [(2*FPG) + TG]*BMI)/(Ln[HDL-C])(1mmol/L=18mg/dl) (29).

### Statistical analysis

Statistical analyses were completed by Empower (R) (www.empowerstats.com) and R-project (version 3.4.3). A two-tailed P value<0.05 indicates statistical significance.

First, all participators were assigned the quartiles of METS-IR. The continuous data under normal distribution were presented in the form of average ± standard deviation, while the skewed distribution was represented by the median (quartile range). One-way analysis and Kruskal-Wallis were utilized for comparing the distribution of normal distribution and skewed distribution between groups. The classified variables were described as numbers (proportions), and comparisons between groups were assessed using the chi-square test.

Subsequently, linear regression analysis was conducted to verify and check the collinearity of variables and report the variance inflation factor (VIF) (31). Variables with VIF larger than 5 were multicollinearity and could not be included in the Cox regression model. Cox proportional hazards model was carried out to analyze the hazard ratio (HR) and 95% confidence interval (CI) for evaluating METS-IR and the risk of new-onset diabetes. The results of three covariate models were demonstrated on the grounds of the recommendations of the STROBE statement to manipulate for possible confounding bias. Covariates with matched hazard ratio change>10% can be added as confounders into the model (32). Crude model: unadjusted, Model I: adjusted for age, gender, smoking status, drinking status, and family history of diabetes, Model II: based on Model I, SBP, DBP, TC, LDL-C, and serum creatinine (SCR) were further adjusted. Further, METS -IR was transformed into a classified variable and trend analysis was calculated for quantifying the stability of the results of regression analysis and observing the nonlinear probability. Moreover, the covariables were converted into the ‘GAM Model’ by the weighted generalized additive model (GAM) to cover the shortage of general linear analysis in the analysis of nonlinearity (33).

Moreover, stratified analysis and interaction testing were applied to analyze the potential effects of METS-IR on incident diabetes in subgroups using multivariable logistic regression models.

The dose-response relationship between METS-IR and incident diabetes was conducted by using a smooth curve fitting and GAM. In the presence of nonlinear correlation, the threshold effect was carried out using a two-piecewise linear regression mode. When the rate between incident diabetes and METS-IR appeared apparent, the inflection point will be calculated automatically by the recursive method.

Furthermore, survival estimates and cumulative diabetes incidence were constructed by the Kaplan-Meier survival analyses, and the log-rank test was used to compute the survival curve functions between METS-IR quartiles.

Additionally, a receiver operating characteristic (ROC) curve was performed to evaluate the performance of METS-IR to predict incident diabetes. Further, ROC was plotted to compare the predictive ability of TG/HDL-C (triglycerides/HDL-c), TγG (Ln[(Glucose*Triglycerides)/2]), TγG-BMI (TyG*BMI) and METS-IR for incident diabetes.

## Result

### Baseline characteristics and univariate analysis of study participants

As shown in **Table 1**, the characteristics of the 116,855 research participators at baseline were described. The total study populations were 62,868 men (53.8%) and 53,987 women (46.2%), and the average age of the research participators was 44.08 ± 12.93 years. At a median follow-up of 3.1± 0.94 years, 2,685 participators (2.3%) had new-onset diabetes. Participants in the higher METS-IR quartile group were older than those in the lower quartile groups (Q1-3). BMI, SBP, DBP, FPG, TC, TG, LDL-C, and SCR increased with the increase of the METS-IR quartile, while HDL-C level decreased (all P values < 0.001). By contrast with participators in quartile 1, subjects in the higher quartiles were more current drinkers and fewer current smokers. No significant differences were found in the Family history of diabetes between the METS-IR quartile group. Univariate linear regression analyses were constructed to examine all significant variables in **Table 2**. And it showed that age, BMI, SBP, DBP, FPG, TC, TG, LDL-C, METS-IR, and family history of diabetes were positively correlated with new-onset diabetes.

**Table 1:** The Baseline Characteristics of participants.

MEST-IR	Q1 (≤33.6)	Q2 (33.6 to ≤38.4)	Q3 (38.4 to ≤43.7	Q4 (43.7to ≤96.7)	*P*-value
**Participants**	29160	29214	29233	29248	
**AGE (years)**	39.653 ± 11.750	43.547 ± 12.623	46.055 ± 13.001	47.047 ± 13.026	<0.001
**GENDER**					<0.001
**Male**	7633 (26.18%)	13580 (46.49%)	18871 (64.55%)	22784 (77.90%)	
**Female**	21527 (73.82%)	15634 (53.51%)	10362 (35.45%)	6464 (22.10%)	
**BMI(kg/m^2^)**	19.642 ± 1.427	22.163 ± 1.291	24.274 ± 1.445	27.298 ± 2.473	<0.001
**SBP(mmHg)**	111.711 ± 14.359	117.090 ± 15.689	121.992 ± 16.117	126.879 ± 16.529	<0.001
**DBP(mmHg)**	69.811 ± 9.451	72.570 ± 10.184	75.924 ± 10.606	79.430 ± 11.146	<0.001
**FPG(mg/dL)**	86.999 ± 9.936	90.318 ± 10.157	92.744 ± 10.824	95.982 ± 11.837	<0.001
**TC(mg/dL)**	86.513 ± 15.782	87.474 ± 16.460	89.558 ± 16.811	90.891 ± 16.942	<0.001
**TG(mg/dL)**	15.528 ± 7.155	20.373 ± 11.038	27.187 ± 16.660	38.885 ± 26.875	<0.001
**HDL-C(mg/dL)**	29.935 ± 5.501	26.371 ± 4.434	24.258 ± 4.196	21.138 ± 4.309	<0.001
**LDL-C(mg/dL)**	48.671 ± 11.930	50.592 ± 12.504	52.416 ± 12.798	52.938 ± 13.239	<0.001
**SCR(mmol/L)**	63.381 ± 13.793	68.624 ± 15.245	73.345 ± 16.106	75.963 ± 15.070	<0.001
**Smoking status**					<0.001
**Never smoker**	747 (2.562%)	1216 (4.162%)	1875 (6.414%)	2834 (9.690%)	
**Ever smoker**	116 (0.398%)	283 (0.969%)	403 (1.379%)	526 (1.798%)	
**Current smoker**	6280 (21.536%)	6213 (21.267%)	6133 (20.980%)	6060 (20.719%)	
**Not recorded**	22017 (75.504%)	21502 (73.602%)	20822 (71.228%)	19828 (67.793%)	
**Drinking status**					<0.001
**Never drinker**	82 (0.281%)	160 (0.548%)	261 (0.893%)	375 (1.282%)	
**Ever drinker**	628 (2.154%)	1176 (4.025%)	1700 (5.815%)	2031 (6.944%)	
**Current drinker**	6433 (22.061%)	6376 (21.825%)	6450 (22.064%)	7014 (23.981%)	
**Not recorded**	22017 (75.504%)	21502 (73.602%)	20822 (71.228%)	19828 (67.793%)	
**Family history of diabetes**					0.743
**NO**	28525 (97.822%)	28551 (97.731%)	28560 (97.698%)	28579 (97.713%)	
**YES**	635 (2.178%)	663 (2.269%)	673 (2.302%)	669 (2.287%)	

Values are n(%) or mean ± SD.

BMI, body mass index; SBP, Systolic blood pressure; DBP, diastolic blood pressure; FPG, fasting plasma glucose; TC, total cholesterol; TG, triglyceride; HDL-C, high-density lipoprotein cholesterol; LDL-C, low-density lipid cholesterol; SCR, serum creatinine.

**Table 2:** The results of univariate analysis.

	Statistics	HR(95%CI)	*P* value
**AGE (years)**	44.079 ± 12.930	1.064 (1.062, 1.067)	<0.0001
GENDER			
**Male**	62868 (53.800%)	Ref	<0.0001
**Female**	53987 (46.200%)	0.496 (0.456, 0.539)	
**BMI(kg/m^2^)**	23.347 ± 3.298	1.222 (1.212, 1.233)	<0.0001
**SBP(mmHg)**	119.424 ± 16.677	1.037 (1.035, 1.039)	<0.0001
**DBP(mmHg)**	74.437 ± 10.975	1.042 (1.039, 1.045)	<0.0001
**FPG(mg/dL)**	91.514 ± 11.208	1.132 (1.129, 1.136)	<0.0001
**TC(mg/dL)**	88.611 ± 16.594	1.016 (1.014, 1.018)	<0.0001
**TG(mg/dL)**	25.502 ± 19.245	1.013 (1.012, 1.013)	<0.0001
**HDL(mg/dL)**	25.422 ± 5.636	0.971 (0.964, 0.977)	<0.0001
**LDL(mg/dL)**	51.156 ± 12.738	1.016 (1.013, 1.019)	<0.0001
**SCR(mmol/L)**	70.339 ± 15.821	1.007 (1.006, 1.008)	<0.0001
**MEST-IR**	39.123 ± 7.393	1.093 (1.089, 1.097)	<0.0001
Smoking status			
**Never smoker**	6672 (5.710%)	Ref	
**Ever smoker**	1328 (1.136%)	0.863 (0.630, 1.181)	0.3620
**Current smoker**	24686 (21.125%)	0.422 (0.361, 0.495)	<0.0001
**Not recorded**	84169 (72.029%)	0.650 (0.570, 0.740)	<0.0001
Drinking status			
**Never drinker**	878 (0.751%)	Ref	
**Ever drinker**	5535 (4.737%)	0.481 (0.324, 0.715)	0.0003
**Current drinker**	26273 (22.483%)	0.515 (0.359, 0.740)	0.0003
**Not recorded**	84169 (72.029%)	0.602 (0.422, 0.858)	0.0050
Family history of diabetes			
**NO**	114215 (97.741%)	Ref	
**YES**	2640 (2.259%)	1.403 (1.148, 1.715)	0.0009

BMI, body mass index; SBP, Systolic blood pressure; DBP, diastolic blood pressure; FPG, fasting plasma glucose; TC, total cholesterol; TG, triglyceride; HDL-C, high-density lipoprotein cholesterol; LDL-C, low-density lipid cholesterol; SCR, serum creatinine; METS-IR: the metabolic score for insulin resistance.CI: confidence, Ref: reference.

### The multivariate analysis between METS-IR and the risk of new-onset diabetes

Firstly, variable collinearity diagnostics were conducted to calculate the VIF for each covariate. Covariates were deemed to exhibit substantial multicollinearity and were ineligible for inclusion in the multivariate Cox regression model if their VIF was more than 5. The results and details were listed in **Table S1**. After that, the outcome of the Cox proportional hazards regression analysis was summarized in **Table 3**. In the Crude model, METS-IR was a positive correlation with incident diabetes (HR= 1.093, 95%CI:1.089 to 1.097, *P* < 0.0001). Compared with the crude mode, HR(95% CIs) for diabetes incidence was 1.083 (1.078 to 1.087) in Model I. Furthermore, the HR for the incident diabetes was 1.077 (95% CI: 1.073, 1.082, *P*<0.0001) in Model II. Remained significant even after the continuous covariates were adopted into the GAM model as curves, and the hazard ratio was 1.085 (95% CI: 1.075 to 1.096, <0.0001), indicating the robustness of the main results. Moreover, when METS-IR was handled as a classified variable into quartiles and using the first quartile as a reference, the trend of incident diabetes increased with METS-IR quartile (*P* for trend<0.001) in the Crude model. In addition, the risk of incident diabetes was increased with Q4 versus Q1 of METS -IR in Model II (HR 6.261[5.189,7.554], *P* for trend<0.001). Consistently, there were significantly stronger associations of the METS-IR with incident diabetes.

**Table 3:** Relationship between METS-IR and the incidence of diabetes in different models.

Variable	Crude model (HR,95%CI,*P*)	Model I (HR,95%CI,*P*)	Model II (HR,95%CI,*P*)	GAM (HR,95%CI,*P*)
**METS-IR**	1.093 (1.089, 1.097) <0.0001	1.083 (1.078, 1.087) <0.0001	1.077 (1.073, 1.082) <0.0001	1.085 (1.075, 1.096) <0.0001
METS-IR (quartile)				
**Q1**	Ref.	Ref.	Ref.	Ref.
**Q2**	2.011 (1.624, 2.490) <0.0001	1.543 (1.245, 1.913) 0.0001	1.470 (1.185, 1.823) 0.0005	1.255 (0.796, 1.720) 0.3286
**Q3**	5.428 (4.491, 6.561) <0.0001	3.542 (2.922, 4.293) <0.0001	3.237 (2.667, 3.927) <0.0001	2.913 (1.954, 4.341) <0.0001
**Q4**	12.158 (10.150, 14.564) <0.0001	7.274 (6.040, 8.760) <0.0001	6.261 (5.189, 7.554) <0.0001	5.713 (3.884, 8.404) <0.0001
***P* for trend**	2.351 (2.248, 2.458) <0.0001	2.046 (1.951, 2.145) <0.0001	1.942 (1.851, 2.038) <0.0001	1.942 (1.757, 2.146) <0.0001

Crude model: we did not adjust other covariants.

Model I: we adjust age, gender, smoking status, drinking status, and family history of diabetes.

Model II: we adjust age, gender, SBP, DBP, TC, LDL-C, SCR, smoking and drinking status, family history of diabetes.

GAM: All covariates listed in model II were adjusted. However, continuous covariates were adjusted as nonlinearity.

Abbreviations: SBP, Systolic blood pressure; DBP, Diastolic blood pressure; TC, total cholesterol; LDL-C, Low-density lipid cholesterol; SCR, serum creatinine; METS-IR: the metabolic score for insulin resistance;GAM, generalized additive model; CI, confidence interval; Ref: reference.

### Kaplan-Meier analysis of diabetes

**Figure 1:**
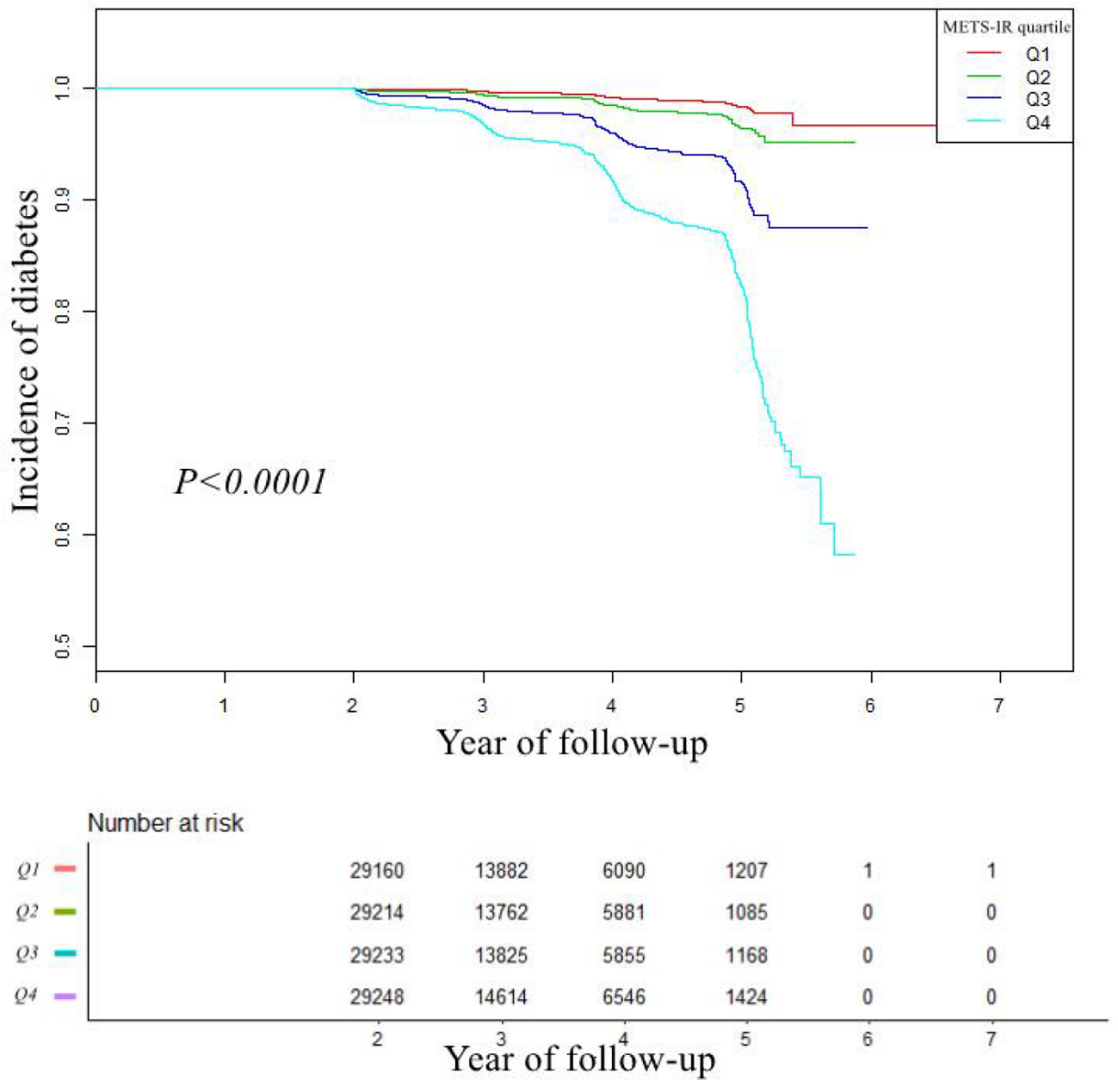
Kaplan–Meier event-free survival curve. Kaplan–Meier analysis of incident of diabetes based on METS-IR quartiles (Log-rank, *P* < 0.0001). Abbreviation: METS-IR: the metabolic score for insulin resistance.

The comparison of cumulative diabetes incidence between the quartiles of baseline METS-IR in the Kaplan-Meier curve was shown in **Figure 1**. Cumulative incidence was the highest in quartile 4 and lowest in quartile1 (log-rank test *P* values < 0.001). It showed that METS-IR Q4 participants had a greater chance of developing incident diabetes than other groups during the follow-up.

### The analyses of the dose-response and threshold effect

**Figure 2:**
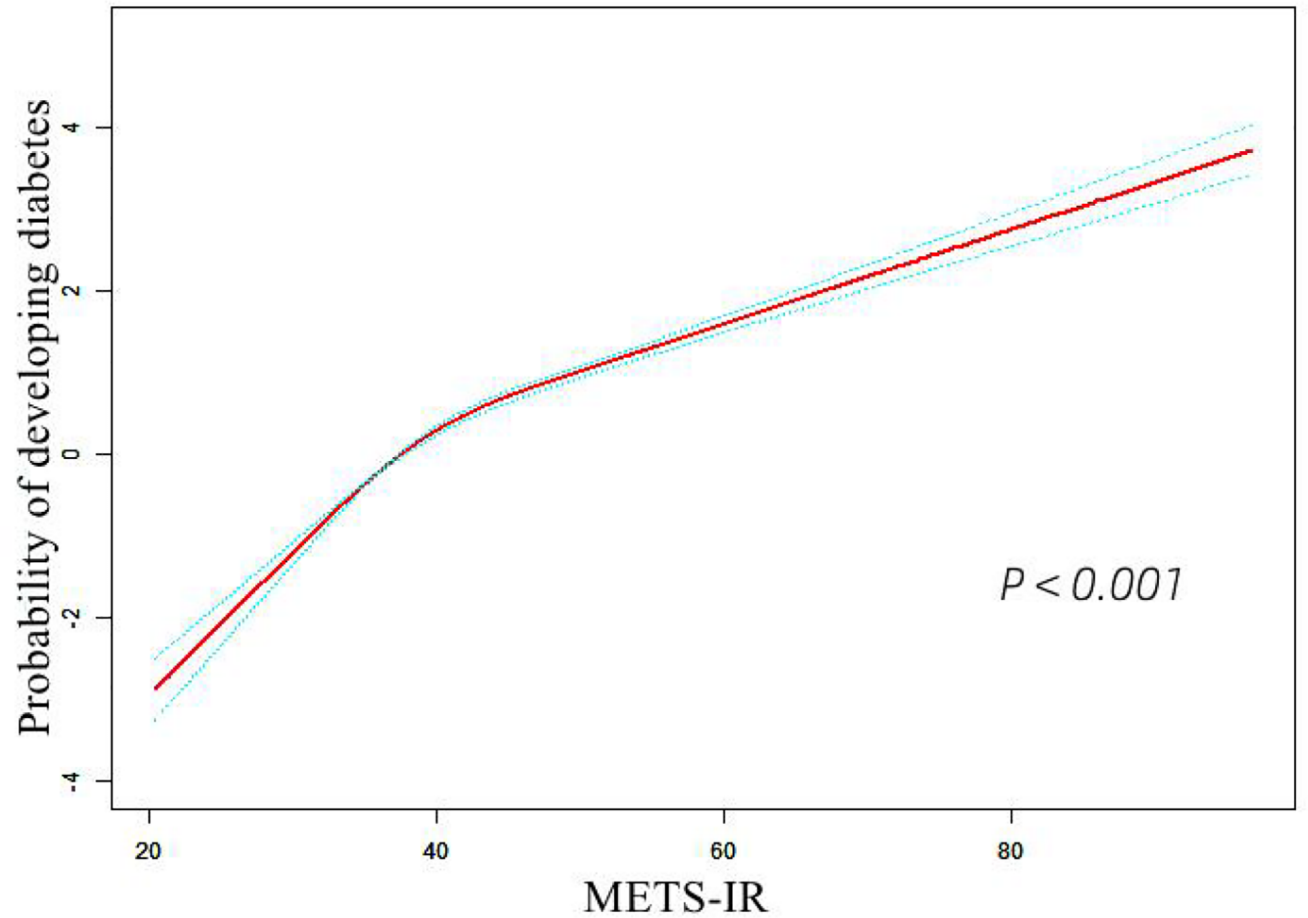
The non-linear relationship between METS-IR and incident of diabetes. Abbreviation: METS-IR: the metabolic score for insulin resistance.

A dose-response study using GAM indicated a non-linear connection between METS-IR and incident diabetes (adjusting age, sex, SBP, DBP, TC, LDL-C, SCR, smoking status, drinking status, and family history of diabetes) in **Figure 2**. We further explored the inflection point of METS-IR was 44.43 (log-likelihood ratio test *P* < 0.001, **Table 4**). When METS-IR < 44.43, the HR was 1.140 (95% CI: 1.126 to 1.154), however, cubic spline smoothing gradually became saturated (HR: 1.051; 95% CI: 1.043 to 1.058) on the right side.

**Table 4:** The result of two-piecewise linear regression model.

	incident of diabetes (HR,95%CI, P)
Fitting model by standard linear regression	1.077 (1.073, 1.082) <0.0001
Fitting model by two-piecewise linear regression	
Inflection point of METS-IR	44.43
≤44.43	1.140 (1.126, 1.154) <0.0001
> 44.43	1.051 (1.043, 1.058) <0.0001
P for log likelihood ratio test	<0.001

We adjusted age, gender, SBP, DBP, TC, LDL-C, SCR, family history of diabetes, smoking and drinking statuses.

Abbreviations: SBP, Systolic blood pressure; DBP, Diastolic blood pressure; TC, total cholesterol; LDL-C, Low-density lipid cholesterol; SCR, serum creatinine; METS-IR: the metabolic score for insulin resistance. CI, confidence interval.

### The results of stratified analyses

In addition, this study conducted a stratified analysis to investigate the effects of potential modifications between METS-IR and incident diabetes, including age, gender, sex, age, BMI, SBP, DBP, FPG, and family history of diabetes. (**Table 5**). Age, BMI, SBP, DBP, and FPG interacted with METS-IR and incident diabetes by interaction tests (all *P*_interaction_ < 0.05). Nevertheless, there was no significant interaction among different stratifications in gender and family history of diabetes. This suggested that the relationship between METS-IR and diabetes mellitus was not affected by gender and family history, and the combination of certain risk factors with METS-IR may enhance its sensitivity.

**Table 5:** Effect size of METS-IR on diabetes in prespecified and exploratory subgroups.

Characteristic	No of participants	HR (95%CI)	P value	P for interaction
**Age(years)**				0.0106
**20 to < 30**	11204	1.091 (1.055, 1.128)	<0.0001	
**30 to < 40**	41985	1.078 (1.064, 1.092)	<0.0001	
**40 to < 50**	27171	1.056 (1.044, 1.067)	<0.0001	
**50 to < 60**	19569	1.025 (1.015, 1.035)	<0.0001	
**≥60**	16926	1.017 (1.005, 1.030)	0.0051	
**Gender**				0.4759
**Male**	62868	1.030 (1.022, 1.039)	<0.0001	
**Female**	53987	1.034 (1.024, 1.044)	<0.0001	
**BMI**				0.0027
**< 18.5**	5991	1.009 (0.878, 1.160)	0.8976	
**≥ 18.5, < 24**	63581	1.052 (1.039, 1.064)	<0.0001	
**≥ 24, < 28**	37151	1.030 (1.018, 1.042)	<0.0001	
**≥ 28**	10132	1.019 (1.007, 1.031)	0.0021	
**SBP**				<0.0001
**<140**	103990	1.039 (1.031, 1.046)	<0.0001	
**≥ 140**	12847	1.011 (1.001, 1.022)	<0.0001	
**DBP**				0.0139
**<90**	106653	1.035 (1.027, 1.042)	<0.0001	
**≥ 90**	10184	1.019 (1.007, 1.032)	<0.0001	
**FPG**				<0.0001
**<100**	93628	1.069 (1.057, 1.081)	<0.0001	
**≥100**	23227	1.023 (1.015, 1.031)	<0.0001	
**Family history of diabetes**				0.2764
**No**	114215	1.032 (1.025, 1.040)	<0.0001	
**Yes**	2640	1.018 (0.993, 1.044)	0.1646	

1:Above model adjusted for age, gender, BMI, SBP, DBP, FPG,and family history of diabetes.

2:In each case, the model is not adjusted for the stratification variable

Abbreviations: BMI, body mass index; SBP, Systolic blood pressure; DBP, diastolic blood pressure; FPG, fasting plasma glucose; CI: confidence.

### The discernibility of METS-IR for diabetes

**Figure 3:**
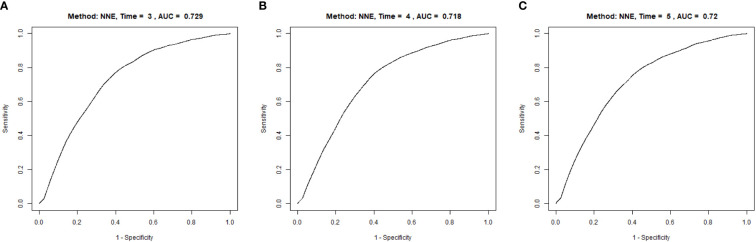
Time-dependent receiver operating characteristic (ROC) curves of METE-IR for diabetes at 3 **(A)**, 4 **(B)** and 5 years **(C)**. METS-IR, the metabolic score for insulin resistance.

**Figure 4:**
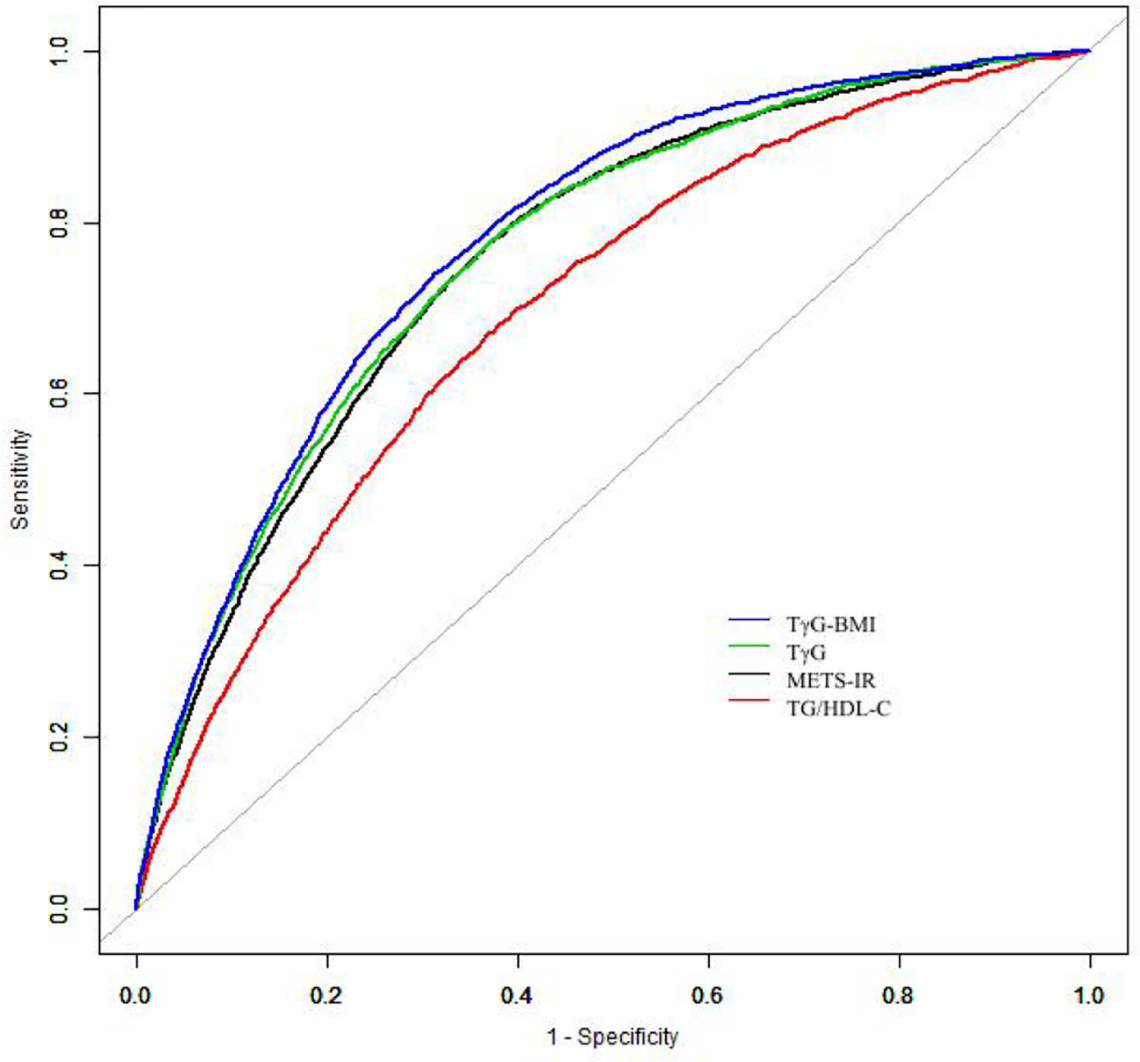
The results of receiver operating characteristics curves. Abbreviations: TG/HDL-C, Triglyceride to high-density lipoprotein cholesterol ratio; TγG index, Triglyceride glucose index; TγG-BMI, TyG*BMI; METS-IR, The Metabolic score of insulin resistance.

A time-dependent ROC analysis was performed to assess the predictive efficacy of METS-IR for incident diabetes at various time nodes (**Figures 3A–C**). The area under the curve (AUC) was 0.729, 0.718, 0.720 at 3, 4, and 5 years, respectively, which revealed a good discriminatory capacity for incident diabetes. In addition, ROC revealed that TG/HDL-C, TγG, TγG-BMI, and METS-IR had AUCs of 0.699, 0.765,0.778, and 0.759, respectively. It seemed that the predictive ability of METS -IR followed by TγG-BMI and was not inferior to TG/HDL-C and TγG (**Figure 4**). And the cut-off points for the prediction of diabetes with them were shown in **Table S2**. Therefore, METS-IR can be used to predict incident diabetes during follow-up in Chinese.

## Discussion

The present large cohort Chinese study demonstrated that there was a positive correlation between METS-IR and the onset of diabetes in Chinese. When adjustments were made for covariates, individuals in the top quartile of METS-IR had a 6.261-fold higher risk of developing diabetes than those in the bottom METS-IR quartile. Meanwhile, the association between METS-IR and incidence diabetes was demonstrated to be nonlinear. Furthermore, the result revealed that the cumulative risk of incident diabetes in Chinese adults increased gradually with the increase of METS-IR. Moreover, ROC analyses suggested the METS-IR had significant discriminatory power for new-onset diabetes at 3 years,4 years, and 5 years. These results indicated that METS-IR can be used to predict the onset of diabetes in healthy individuals during follow-up.

Non-insulin-based metabolic indicators used to evaluate IR have developed into an easier and cost-effective alternative method, which is more suitable for epidemiological studies. The previously widely accepted non-insulin-based IR indicators commonly included FPG, parameters of lipids, and indices of obesity, such as TyG, TyG-BMI, and TG/HDL-C (34–36). Likewise, the METS-IR is a simple and economical indicator that combines FPG, lipid profile, and obesity index. It is reported that TyG (23, 24, 37, 38), TyG-BMI (27, 28), and TG/HDL-C (25) were correlated with the risk of diabetes positively. Consistent with present study, previous studies (29, 39, 40) also identified that there was a positive correlation between METS-IR and incident diabetes. Additionally, a prospective cohort study by Chavolla et al. also proved that METS-IR was superior to the TγG index and TG/HDL ratio in diagnostic performance, but there was no significant difference between METS-IR and TγG-BMI index (29). However, ROC analysis in this research was observed that TG/HDL-C, TγG, TγG-BMI and METS-IR had AUCs of 0.699, 0.765,0.778, and 0.759, respectively, it revealed that the discriminatory power of METS-IR was still superior to TG/HDL ratio, but not TγG. Moreover, Chavolla et al. proved that the effect between METS-IR and diabetes was modulated by age (29). An epidemiological study conducted by 12,107 Chinese participants and subgroup analyses also consistently confirmed that significant associations remained between gender, age, and FPG level in subgroup analyses (39). Interestingly, stratified interaction analysis in this study also found differences in the influence of METS-IR and diabetes among age and baseline FPG subgroups, but there was no interaction observed in the subgroup of gender, suggesting that the correlation between METS-IR and diabetes was robust among men and women. Notably, this study also conducted the dose-response analysis between METS-IR and diabetes and showed that the probability of diabetes gradually increased with the increase of METS-IR, which is consistent with results from a cohort of 12,107 rural Chinese participants (39) and a cohort study of 12,290 non-obese Japanese adults (40). Nonetheless, this study provided additional information that there was a positive correlation between METS-IR and the incidence of DM with a saturation effect, and the inflection point of METS-IR was calculated to be 44.43. When METS-IR ≤44.43, the risk of diabetes increased rapidly with METS-IR, the effect size was 1.140 (95% CI: 1.126-1.154, P < 0.0001); while METS-IR>44.43, the tendency gradually saturated compared with the left side of the inflection point (HR=1.051,95%CI: 1.043,1.058 P<0.0001). A possible explanation for the conflicting results may be the differences ​in participant selection and covariables, and further studies are needed to check on the result.

The underlying mechanism of METS-IR associated with diabetes has yet to be elucidated. IR has played its pathological mechanism before the onset of diabetes (41). The content of reactive oxygen species (ROS) increases with the increase of blood glucose, which may have a toxic reaction on β-cells, leading to pancreatic β-cell dysfunction, and then causing diabetes (42, 43). During hyperinsulinemia, the efficiency of insulin signaling is inhibited, resulting in reduced glucose uptake from the blood, which increases the risk of diabetes (44). Consecutively, hyperglycemia can induce excessive peroxide production, which eventually leads to impaired insulin secretion and insulin resistance by promoting multiple oxidative stress pathways (45).There is growing evidence about dyslipidemia is a cause of IR (46, 47). Diabetes is a progressive disease, and a study on animal models of diabetes showed β-cell apoptosis accompanied by long-term hyperglycemia/hyperlipemia (glucolipotoxicity) (48). Previous literature indicated that fat accumulation is related to IR, which may provoke metabolic disturbances in the liver, and further affect the homeostasis of blood glucose and lipid (49–51). Adipose tissue contributes to metabolic risk and, in addition to the effects of body mass index, is associated with elevated blood glucose and lower HDL-C (52, 53). Hypertriglyceridemia contributes to a corresponding increase in free fatty acid (FFA) levels, which may impair insulin signaling and induce tissue oxidative stress, giving rise to IR in bone and liver (53, 54). Simultaneously, lower HDL-C reduces its anti-inflammatory effects and its inhibitory effect on LDL-C oxidation (55). Besides, IR is affected by many factors, obesity index is also an important factor in IR (56), which can occur even in people with a normal BMI (57). And BMI has been shown to have a strong association with prediabetes and diabetes in many studies (58–61). Therefore, as one of the important components of the METS-IR model, the obesity index may have a significant impact on the prediction of diabetes by METS-IR. Chavolla and his colleagues found that subjects with high METS-IR index showed increased visceral fat and fasting insulin levels (29). At the same time, in our prospective study, higher levels of BMI, FPG, TC, TG, and LDL-C were observed at higher baseline METS-IR levels, and persons in higher baseline METS-IR levels had higher cumulative incidence of diabetes, which supported METS-IR can be applied to forecast the prevalence of diabetes.

There were still potential limitations that should be considered in this study. First, the occurrence of diabetes was diagnosed only by FPG and self-report in this study, without the use of the 2-hour oral glucose tolerance test (OGTT) and glycated hemoglobin (HbA1c), which may underestimate the incidence of diabetes in the study. However, due to the operation complexity of OGTT, it is not feasible for large cohorts and similar limitations have been observed in previous large population studies (40). Second, due to the limitations of the original cohort data, we could not include several potential confounders and biochemical indicators such as diet, exercise, and plasma insulin. In particular, the lack of plasma insulin limited the ability to explore the concordance between METS-IR and HOMA-IR, and patients with diabetes could not be classified in the study because insulin testing is not a routine physical examination, especially in large epidemiological studies. Additionally, there was a lack of data on the dynamic changes of METS-IR during the follow-up survey, so its correlation with diabetes could not be evaluated in this study. Third, the study population was all Chinese, which may limit the extrapolation of such results to other ethnic groups. Despite these limitations, China has a large population of diabetics (3, 4), and the results of this study are still representative.

Although our study had limitations, there were still several advantages. Compared with previous similar studies (29, 39, 40), this study was a large sample size and a multicenter study. Furthermore, the study also conducted a sensitivity analysis by handling METS -IR as a categorical variable and continuous variable to assess the stability of the result.

## Conclusion

This study provides additional evidence supporting that METS-IR was connected with incident diabetes in the cohort of Chinese, and there is a non-linear relationship between METS-IR and diabetes. Meanwhile, METS-IR had a good discriminative ability for incident diabetes. METS-IR may be a reliable alternative method for predicting the risk of diabetes in epidemiological investigations.

## Data availability statement

The original contributions presented in the study are included in the article/**Supplementary Materials**. Further inquiries can be directed to the corresponding author.

## Ethics statement

The studies involving human participants were reviewed and approved by Rich Healthcare Group. The patients/participants provided their written informed consent to participate in this study.

## Author contributions

KF and ZC contributed to the study concept and design, researched and interpreted the data and drafted the manuscript. CH, ZZ and YZ performed the statistical analysis. MX, YT and LF contributed to the discussion. ZC drafted the Manuscript and KF edited the manuscript. All authors read and approved the final the manuscript. All authors contributed to the article and approved the submitted version.
