# Supplementary material for: A nonlinear associations of metabolic score for insulin resistance index with incident diabetes: A retrospective Chinese cohort study
Source: Front Clin Diabetes Healthc. 2023 Jan 12;3:1101276. doi: 10.3389/fcdhc.2022.1101276 (PMC10012088; doi:10.3389/fcdhc.2022.1101276)
Supplement: Supplementary file 1 [file DataSheet_1.docx]

**Table S1.** Collinearity diagnostics steps

|  | Step 1 |
| --- | --- |
| Age | 1.2 |
| Gender | 2.2 |
| SBP | 2.2 |
| DBP | 2.1 |
| TC | 4.3 |
| LDL-C | 5.6 |
| SCR | 2 |
| METS-IR | 1.3 |
| Smoking status | 3 |
| Drinking status | 3 |
| Family history of diabetes | 1 |

Abbreviations: SBP, Systolic blood pressure; DBP, Diastolic blood pressure; TC, Total cholesterol; LDL-C, Low-density lipid cholesterol; SCR, serum creatinine. METS-IR: the metabolic score for insulin resistance.

**Table S2.** Effect of TG/HDL-C, TγG, TγG-BMI and METS-IR for the risk of diabetes

| Variable | AUC(95%CI) | Cut-off point | Specificity | Sensitivity |
| --- | --- | --- | --- | --- |
| TG/HDL-C | 0.699(0.689-0.709) | 1.025 | 0.631 | 0.670 |
| TγG | 0.765(0.757-0.774) | 7.025 | 0.629 | 0.778 |
| TγG-BMI | 0.778(0.770-0.787) | 174.575 | 0.688 | 0.739 |
| METS-IR | 0.759(0.750-0.768) | 40.855 | 0.633 | 0.773 |

Abbreviations: TG/HDL-C, Triglyceride to high-density lipoprotein cholesterol ratio; TγG index, Triglyceride glucose index; TγG-BMI, TyG*BMI;METS-IR, The Metabolic score of insulin resistance.
